# An Integrated Zero-Trust and Real-Time Detection Scheme for DDoS Protection in 5G IoT Systems

**DOI:** 10.3390/s26113479

**Published:** 2026-06-01

**Authors:** Yu-Yong Luo, Chia-Hsin Cheng, Yu-Run Lian, Yung-Fa Huang, Cheng-Hsiung Hsieh

**Affiliations:** 1 Department of Electro-Optical Engineering, National Formosa University, Yunlin 632301, Taiwan; 30925120@gm.nfu.edu.tw; 2 Department of Electrical Engineering, National Formosa University, Yunlin 632301, Taiwan; advcomm@gs.nfu.edu.tw; 3 Department of Information and Communication Engineering, Chaoyang University of Technology, Taichung 413310, Taiwan; 4 Department of Computer Science and Information Engineering, Chaoyang University of Technology, Taichung 413310, Taiwan; chhsieh@cyut.edu.tw

**Keywords:** 5G IoT, zero-trust-based permission-control mechanism, distributed denial-of-service (DDoS), long short-term memory (LSTM), support vector machine (SVM)

## Abstract

This study presents a laboratory-scale prototype that integrates a zero-trust-based permission-control mechanism with real-time DDoS traffic detection in a 5G NSA IoT testbed. The proposed system was evaluated under three controlled traffic conditions: normal traffic, TCP SYN flood traffic, and UDP flood traffic. Packet-level data were collected from the experimental testbed and used to train and compare LSTM and SVM classifiers. Under the evaluated conditions, the LSTM model achieved the highest accuracy of 99.56%, outperforming the best SVM result of 93.20%. The selected LSTM detector was further deployed in the edge-computing pipeline and correctly identified the three tested traffic conditions during real-time operation. After malicious traffic was identified, the permission-control mechanism updated the corresponding authorization status, generated an alert, and restricted suspicious communication within the testbed. These results demonstrate the feasibility of linking traffic detection with authorization adjustment in a controlled 5G NSA IoT prototype. The findings should not be interpreted as a general validation against all DDoS variants or large-scale commercial 5G IoT deployments.

## 1. Introduction

### 1.1. Background and Motivation

The rapid development of fifth-generation (5G) mobile networks and Internet of Things (IoT) technologies has significantly reshaped modern connectivity and device deployment. With high data rates, ultra-low latency, and support for massive machine-type communications, 5G provides a key infrastructure for large-scale IoT applications, including smart cities, industrial automation, environmental monitoring, and intelligent transportation systems [1,2,3,4,5]. Compared with traditional wired infrastructures, 5G-enabled IoT environments offer greater deployment flexibility and support a much larger number of heterogeneous and resource-constrained devices. However, this expanded connectivity also enlarges the cybersecurity attack surface and increases the difficulty of protecting IoT devices from malicious activities [1,4,5].

Among the major security threats in such environments, distributed denial-of-service (DDoS) attacks remain one of the most serious challenges. In a DDoS attack, compromised devices are coordinated to send excessive malicious traffic to a target service or network resource, thereby exhausting bandwidth, computational capacity, or protocol state resources and preventing legitimate access. This threat is particularly severe in IoT environments because many IoT devices have limited computing capabilities, simplified security mechanisms, and infrequent maintenance, making them attractive targets for botnet recruitment [5,6]. Once a large number of insecure devices are connected through high-capacity 5G networks, the scale and propagation speed of DDoS attacks may increase substantially. Therefore, developing effective defense mechanisms for 5G IoT environments has become an important and urgent research issue [5,6].

### 1.2. Related Work and Research Gap

Conventional cybersecurity approaches, such as perimeter-based filtering, static access control lists, and signature-based intrusion detection, still play an important role in network defense. However, these methods are often insufficient for dynamic 5G IoT scenarios characterized by frequent device mobility, rapidly evolving traffic patterns, and weakened trust assumptions based solely on network location [7,8,9,10,11,12]. In response, Zero Trust Architecture (ZTA) has emerged as a promising security paradigm in which no user, device, or service is trusted by default. Instead, ZTA emphasizes continuous verification, least-privilege access enforcement, and context-aware authorization for every interaction, regardless of origin [7,8,9,10,11,12]. Recent studies have explored the application of zero-trust principles in 5G, mobile edge computing, and beyond-5G environments [9,10,11,12]. Nevertheless, access control mechanisms alone cannot effectively mitigate ongoing network attacks without complementary real-time threat detection capabilities.

In parallel, machine learning and deep learning techniques have been widely investigated for attack detection because they can identify anomalous traffic patterns more effectively than purely rule-based methods [13]. In particular, sequence-aware models are attractive for network traffic analysis because packet behavior and attack evolution often exhibit strong temporal dependencies [14]. Long Short-Term Memory (LSTM) networks, as a recurrent neural network architecture designed to capture long-range sequential patterns, have demonstrated strong potential in traffic classification and intrusion detection tasks [14,15,16]. Support Vector Machine (SVM), on the other hand, remains a widely used baseline classifier in network security research because of its effectiveness in feature-based classification problems [17]. Although previous studies have addressed traffic-based intrusion detection and zero-trust access control separately, fewer studies have integrated real-time traffic detection with dynamic authorization enforcement in practical 5G non-standalone (NSA) IoT environments. This gap is important because detection without enforceable access control may fail to contain attacks, whereas access control without timely detection may be insufficient for rapidly evolving threats.

Existing studies on DDoS defense in IoT and 5G environments can be broadly divided into two lines of work. One line emphasizes traffic classification and anomaly detection using machine learning or deep learning models, with attention directed toward improving classification accuracy under different attack conditions. The other line focuses on access control, trust management, and zero-trust-based policy enforcement in distributed network environments. Although both lines have produced valuable results, they are often studied at different layers of the defense pipeline. In many cases, detection results remain isolated from enforcement actions, whereas access control mechanisms operate without direct integration with real-time traffic intelligence. This separation limits the ability of existing approaches to contain ongoing attacks immediately after abnormal behavior is identified.

To provide a clearer and more balanced comparison, Table 1 summarizes representative studies related to DDoS traffic detection, zero-trust-based access control, and 5G/IoT security deployment. In addition to comparing technical focuses, the table also identifies the evaluation scope and limitations of each study. This comparison helps position the present work as a laboratory-scale prototype validation, rather than a complete large-scale 5G IoT security solution.

As shown in Table 1, existing studies [7,10,18,19,20,21,22,23] provide valuable foundations for traffic-based attack detection and zero-trust-oriented access control. Detection-oriented studies generally focus on improving classification accuracy under specific datasets or network environments, but their detection results are not always connected to enforceable containment actions. Zero-trust-oriented studies provide useful architectural and policy concepts, but real-time packet-level DDoS detection is often not their primary focus. Compared with these studies, the present work addresses a narrower but operationally connected problem: whether packet-level DDoS detection can be linked to authorization adjustment in a controlled 5G NSA IoT testbed.

However, the scope of this work is intentionally limited. The experiments were conducted on a small laboratory-scale testbed and evaluated only normal traffic, TCP SYN flood traffic, and UDP flood traffic. The implemented permission-control mechanism includes registration, authentication, permission-table update, alert notification, and communication restriction, but it does not implement a complete zero-trust architecture with risk-scored policy decisions, micro-segmentation, or large-scale continuous trust evaluation. Therefore, the contribution of this study should be interpreted as prototype-level feasibility validation rather than full deployment verification.

**Figure 1:**
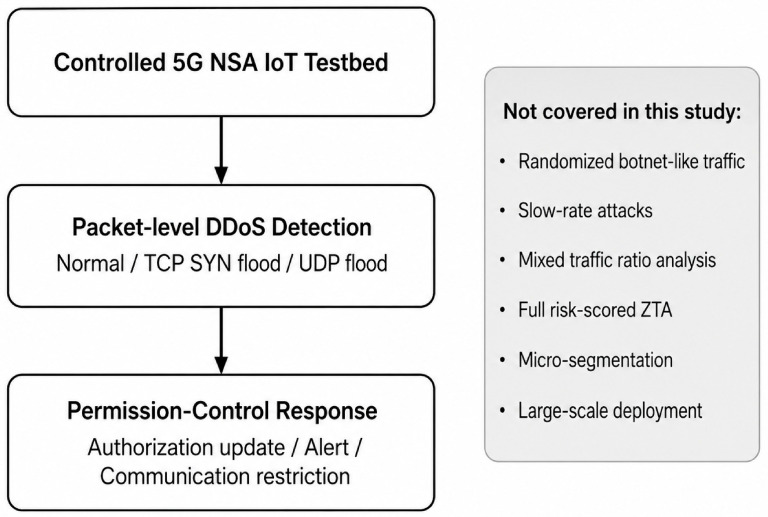
Scope of the proposed laboratory-scale prototype and limitations of the current validation.

To clarify the positioning of this work and avoid overstating the contribution, Figure 1 summarizes the validation scope of the proposed laboratory-scale prototype and the limitations of the current study. The current study focuses on prototype-level functional validation. Within this scope, the proposed framework implemented a detection-to-authorization workflow and demonstrated that the deployed detector could trigger permission updates and communication restriction under the evaluated traffic conditions. As summarized in Figure 1, the validation was limited to a controlled laboratory-scale 5G NSA IoT testbed and three traffic conditions: normal traffic, TCP SYN flood traffic, and UDP flood traffic. Therefore, the reported LSTM accuracy of 99.56% should be interpreted only within these evaluated conditions and should not be regarded as evidence of general robustness against all DDoS variants.

### 1.3. Research Objective and Contributions

The objective of this study is to evaluate a laboratory-scale prototype that links real-time DDoS traffic detection with zero-trust-based permission control in a 5G NSA IoT testbed. The proposed system is not intended to provide a complete DDoS defense solution for commercial-scale 5G IoT deployments. Instead, it focuses on validating whether traffic classification results can be translated into authorization updates, alert notifications, and communication restrictions within a controlled experimental environment.

For empirical evaluation, two detection models, namely LSTM and SVM, were implemented and compared using packet-level traffic traces collected from the 5G NSA IoT testbed. The evaluation covered three traffic classes: normal traffic, TCP SYN flood traffic, and UDP flood traffic. Under these evaluated conditions, the LSTM-based detector achieved an accuracy of 99.56%. The selected detector was then deployed in the real-time edge-computing pipeline to verify whether detection outputs could trigger permission adjustment and communication restriction during controlled testbed operation.

The contributions of this study are limited to the following aspects.

(1) A laboratory-scale 5G NSA IoT testbed was constructed to evaluate the integration of packet-level DDoS detection and permission control.

(2) LSTM and SVM were compared under three controlled traffic classes: normal traffic, TCP SYN flood traffic, and UDP flood traffic.

(3) A prototype response workflow was demonstrated, in which detection results triggered authorization updates, alert notification, and communication restriction within the testbed.

## 2. Materials and Methods

### 2.1. Overall Framework

**Figure 2:**
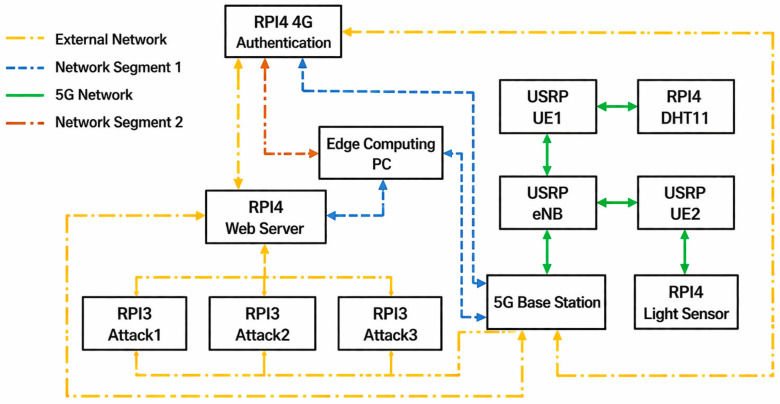
Experimental architecture diagram.

This study proposes an integrated security framework for 5G non-standalone (NSA) Internet of Things (IoT) environments that combines a zero-trust-based permission-control mechanism with real-time distributed denial-of-service (DDoS) attack detection. The framework comprises a 5G NSA communication subsystem, IoT end devices, a zero-trust authentication and authorization server, and a real-time traffic analysis module deployed on an edge-computing platform. The architecture is divided into four segments: the 5G network, the external network, network segment 1, and network segment 2. Network segment 1 handles UE authorization verification and forwards captured packet traffic to the edge node, while Network segment 2 delivers abnormality reports from the edge node to the authentication server for permission updates, as shown in Figure 2.

IoT devices periodically transmit sensing data through the 5G network to a web server, with packet-level traffic intercepted and forwarded to the edge node for attack detection. Upon identification of malicious behavior, the detection result is transmitted to the authentication server, which dynamically updates device permissions according to the zero-trust policy. This design enables simultaneous real-time attack identification and adaptive access control to limit attack propagation.

In the present study, the attacker is assumed to control one or more compromised IoT devices capable of generating TCP SYN flood or UDP flood traffic toward the target service. The authentication server and the edge-computing node are treated as trusted components of the proposed framework. Packet-level traffic captured from the communication path is forwarded to the edge node for real-time analysis, and suspicious traffic is reported to the authentication server for permission updates. Under this design, attack containment is achieved through the combined operation of traffic detection and dynamic authorization control.

This assumption defines the boundary of the current threat model. The present prototype focuses on detecting compromised IoT devices and restricting their communication permissions after abnormal traffic is identified. Compromise of the authentication server or edge-computing node is outside the scope of the current implementation. If either trusted component is compromised, the attacker may manipulate detection outputs, authorization records, or enforcement decisions, thereby undermining the containment mechanism. In practical deployment, these components should be protected through system hardening, secure communication channels, access control, integrity monitoring, audit logging, and redundancy. A more complete design should also include mechanisms for verifying the trustworthiness of the edge node and the authentication server themselves.

### 2.2. Experimental Environment and Materials

**Figure 3:**
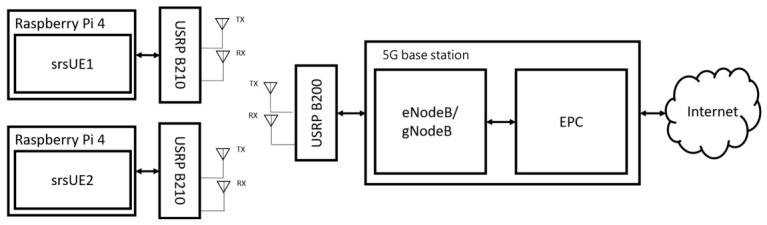
Experimental 5G NSA architecture.

The testbed was constructed using eight Raspberry Pi devices, two computers, and three USRP devices, specifically four Raspberry Pi 3B boards, four Raspberry Pi 4B boards, one edge-computing PC, one 5G base-station PC with EPC and eNB functions, two USRP B210 units, and one USRP B200 unit (Figure 3). Device specifications are summarized in Table 2. The 5G NSA subsystem was implemented using srsRAN in an NSA testbed configuration [24].

The experimental environment was configured as a controlled laboratory-scale 5G NSA IoT testbed for validating the functional feasibility of packet capture, traffic generation, real-time detection, and authorization update. The purpose of this setup was not to reproduce the scale, traffic diversity, or operational complexity of commercial 5G IoT deployments. Therefore, the results reported in this study should be interpreted as prototype-level validation rather than evidence of scalability to large-scale networks.

### 2.3. Zero-Trust Authentication and Authorization Mechanism

**Figure 4:**
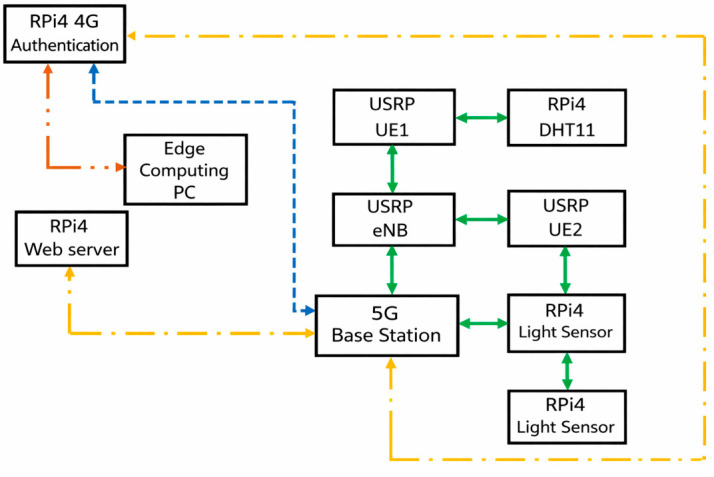
Zero Trust authentication architecture.

**Figure 5:**
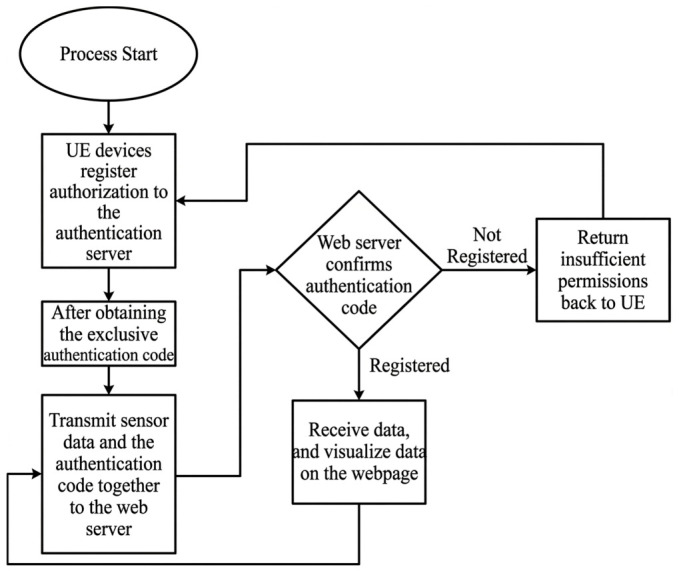
Authentication workflow.

A zero-trust authentication and authorization mechanism was implemented to ensure no device was trusted by default. All IoT devices were required to complete registration and authentication before accessing protected services (Figure 4). Unregistered devices are restricted from normal service communication. After successful registration, devices were granted permissions according to predefined system policies (Figure 5).

The authentication server maintains a permission table for access control decisions. Upon receiving a suspicious traffic report from the edge node, the server updates the corresponding authorization record through a dedicated communication path. This enables dynamic restriction of malicious devices and supports adaptive authorization during attacks.

### 2.4. Real-Time Detection Architecture

**Figure 6:**
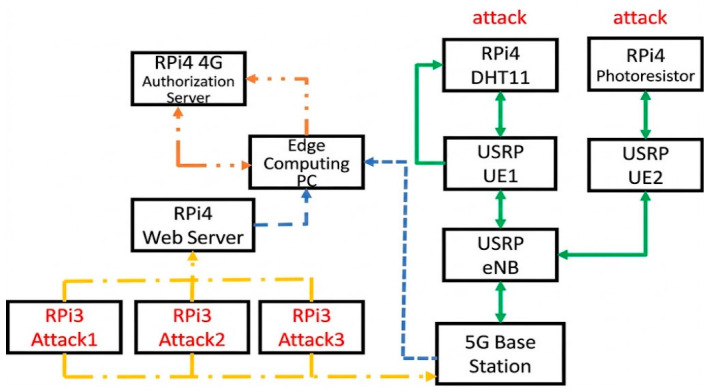
Real-time detection architecture.

**Figure 7:**
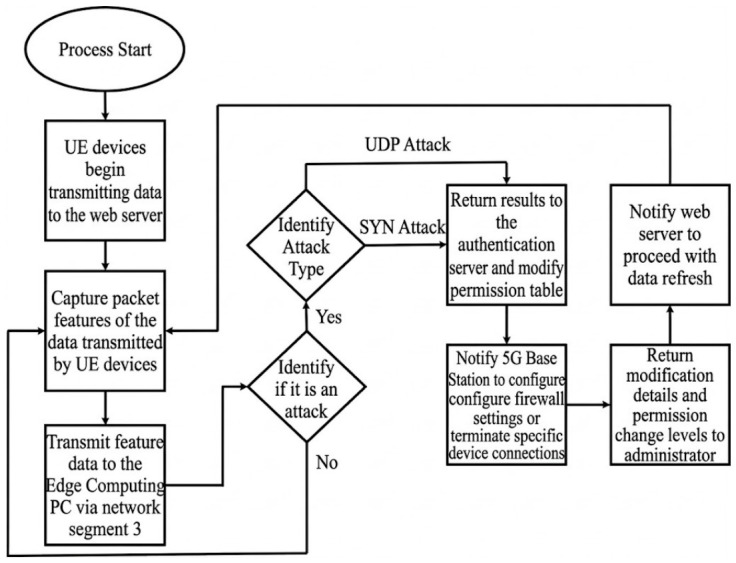
Detection and response workflow.

The real-time detection architecture intercepts traffic from the 5G communication path and couples analysis with authorization control (Figure 6). Packet data are captured near the 5G base station and transmitted via a dedicated local network to the edge-computing PC for classification. Detection results are forwarded to the authentication server for permission updates (Figure 7).

The detection pipeline performs packet-level analysis and classifies traffic into normal, TCP SYN flood, or UDP flood categories. The resulting label determines whether the device retains original permissions or faces restrictions via the zero-trust controller.

### 2.5. Traffic Generation and Packet Capture

**Figure 8:**
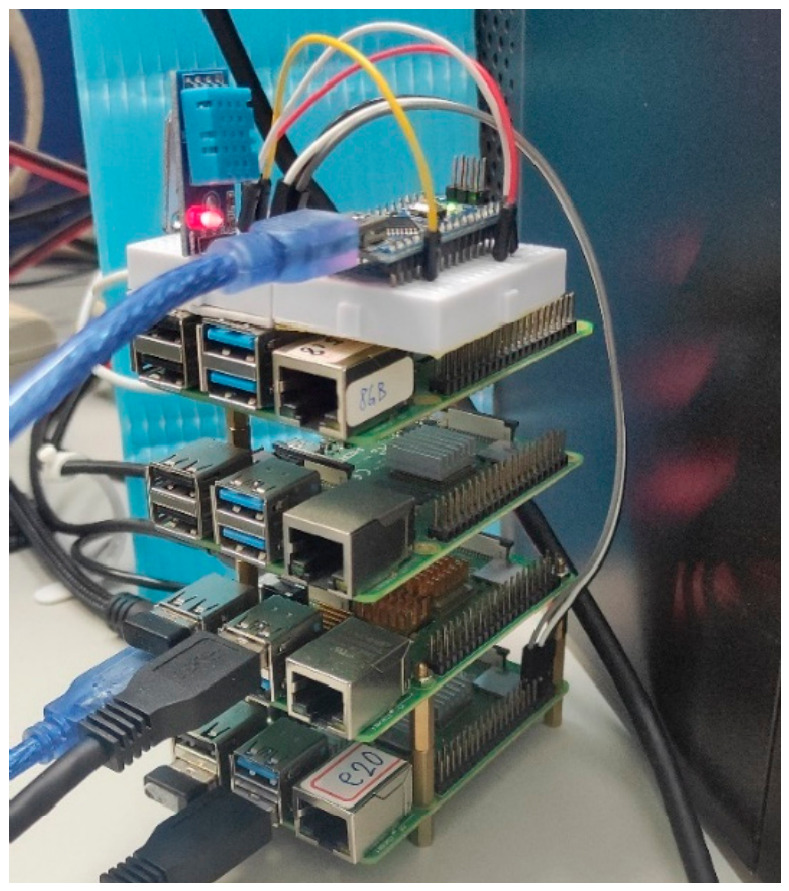
Data collection devices in the 5G IoT environment.

**Figure 9:**
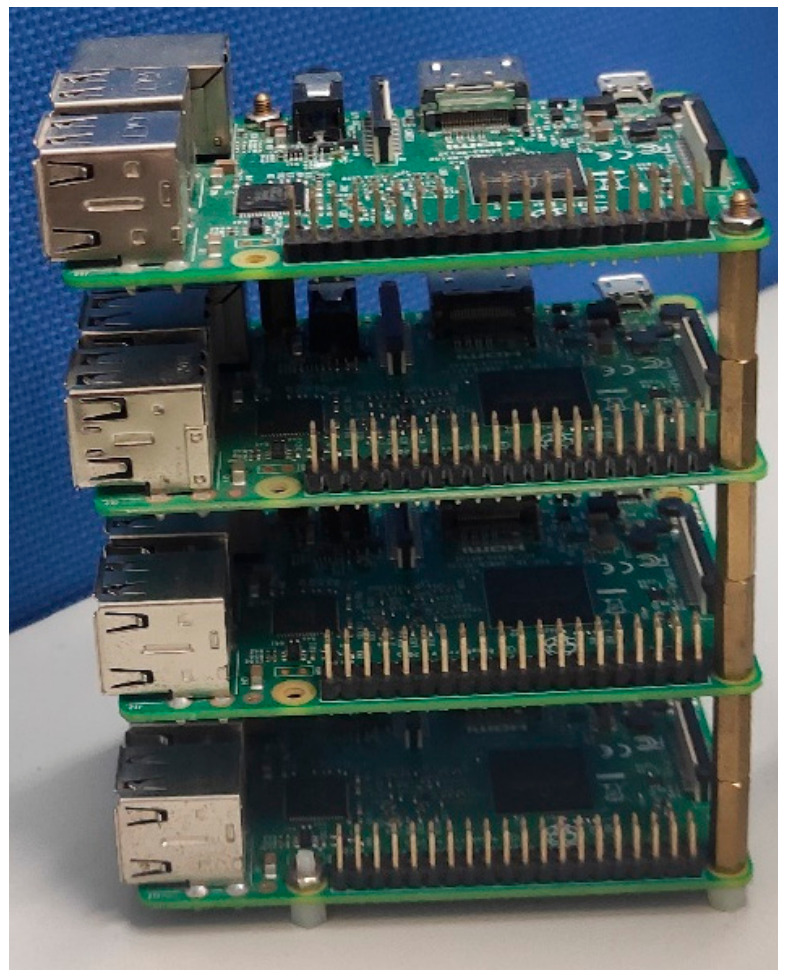
Raspberry Pi devices used to emulate distributed attack sources.

Two Raspberry Pi 4 devices served as 5G IoT sensing nodes, collecting environmental and system data (temperature, humidity, brightness, CPU/memory usage) and transmitting via HTTP POST to the web server every 8 s (Figure 8). Four Raspberry Pi 3 devices emulated distributed attack sources using hping3 for TCP SYN flood and UDP flood [25] (Figure 9, Table 3).

Packet capture used Tshark (command-line Wireshark) [26]. Each traffic condition (normal, SYN attack, UDP attack) was observed for 15 min.

### 2.6. Feature Extraction and Dataset Construction

The dataset consisted of packet-level records captured from the 5G NSA testbed. Each record contained 18 features, namely frame.time, ip.src_host, ip.dst_host, tcp.srcport, tcp.dstport, udp.srcport, udp.dstport, frame.len, tcp.flags, tcp.seq, tcp.ack, tcp.len, udp.length, tcp.stream, udp.stream, cpu_used, mem_used, and label. The class labels were defined as 0 for normal traffic, 1 for TCP SYN flood traffic, and 2 for UDP flood traffic.

The selected features covered packet timing, source and destination information, transport-layer port numbers, frame length, TCP control-related attributes, UDP packet length, stream identifiers, and device-side resource usage. This feature set was intended to preserve both protocol-level traffic behavior and device operating conditions under normal and attack scenarios. For the SVM model, the timestamp field was converted into numerical form, and non-numeric attributes were transformed into machine-readable representations before feature standardization. For the LSTM model, categorical attributes were encoded using OneHotEncoder, and sequences of 10 consecutive records were used to predict the class of the 11th record. This sequence construction allowed the model to capture short-term temporal dependencies in packet evolution across consecutive traffic records.

The selected features were intended to reflect both packet-level protocol behavior and device-side operating conditions. TCP-related fields, such as flags, sequence values, acknowledgment behavior, and stream identifiers, were expected to provide useful cues for identifying SYN flood traffic. UDP-related fields, including port variation and packet length, were relevant to distinguishing UDP flooding behavior. The inclusion of cpu_used and mem_used was intended to preserve device-side status information under both benign and attack conditions, thereby complementing the network-side packet features.

### 2.7. Detection Models and Parameter Settings

Two detection models were implemented and compared in this study: Support Vector Machine (SVM) and Long Short-Term Memory (LSTM). SVM was used as the baseline classifier, whereas LSTM was adopted to capture the sequential characteristics of packet-level traffic. For SVM, the penalty parameter *C* was evaluated at 1, 10, and 100, while the gamma parameter γ was tested at 10, 100, and 1000. For LSTM, three learning rates (0.00001, 0.0001, and 0.001) and three hidden sizes (1024, 2048, and 3072) were examined. In addition, timestamp transformation, categorical encoding, and sequence construction were applied, and 10 consecutive records were used to predict the 11th record. The performance comparison of the two models under these parameter settings is presented in Section 3.1.

SVM was adopted as a baseline because it remains a widely used classifier for traffic analysis under structured feature representations. By contrast, LSTM was selected to capture the short-term temporal dependencies inherent in consecutive packet records. This distinction was particularly relevant in the present study, where attack traffic was expected to exhibit sequential rather than purely isolated feature patterns.

The two models were selected for complementary purposes rather than to provide an exhaustive comparison of all possible classifiers. SVM was used as a conventional machine-learning baseline because it is commonly adopted for structured feature-based intrusion detection and provides a reference point for non-sequential classification. LSTM was selected because the packet records in this study were processed as short sequences, and flooding attacks may exhibit temporal patterns across consecutive packets. However, the use of LSTM also introduces limitations. Its performance depends on the quality, representativeness, and labeling consistency of the training data, and the model may not generalize well to unseen attack variants that differ substantially from the training traffic. Therefore, the reported LSTM accuracy should be interpreted within the evaluated normal, TCP SYN flood, and UDP flood traffic conditions.

### 2.8. Scalability and Latency Evaluation Scope

The current prototype was designed to verify the functional linkage between packet-level traffic detection and authorization update in a controlled 5G NSA IoT testbed. The edge-computing PC was used to execute the trained detector and forward abnormality reports to the authentication server. However, this study did not conduct large-scale stress testing with hundreds or thousands of IoT devices, nor did it benchmark maximum throughput under large concurrent traffic loads. Therefore, the current results do not support the claim that the edge-computing PC can directly handle 1000+ devices in a commercial deployment.

In this prototype, the response workflow consists of four stages: packet capture, edge-side classification, abnormality reporting, and authorization-table update. Although the experiments confirmed that this workflow could trigger permission restriction after attack identification, the exact detection-to-containment delay was not measured as an independent quantitative metric. A complete latency evaluation would require timestamping each stage, including the time of packet capture, model inference, report transmission, authorization update, and enforcement verification. This issue is treated as a limitation of the present study and will be addressed in future work using larger traffic loads and repeated latency measurements.

It should be noted that the Raspberry Pi devices in this testbed were used as sensing nodes and emulated attack sources, whereas the edge-computing function was executed on a dedicated PC equipped with an Intel i7-10700 CPU and 16 GB memory. Therefore, the current implementation does not evaluate the feasibility of running the detection module on resource-constrained Raspberry Pi-based edge nodes.

## 3. Results

### 3.1. Comparison of Detection Models

The comparative performance of the two detection models is summarized in Table 4 and Table 5. Among the SVM configurations, the highest accuracy was 93.20%, achieved when the gamma parameter was set to 10, and the penalty parameter C was set to 10 or 100 (Table 4). In contrast, the LSTM model consistently outperformed SVM and achieved a maximum accuracy of 99.56% when the learning rate was set to 0.001, and the hidden size was 2048 or 3072 (Table 5). These results indicate that the LSTM model was more suitable for packet-level DDoS traffic classification in the 5G NSA IoT testbed. Accordingly, the LSTM model was selected for deployment in the real-time detection module.

To present the comparison more clearly, Table 6 summarizes the best performance achieved by the two classifiers. Under the tested parameter settings, the LSTM model outperformed SVM by 6.36 percentage points in accuracy. This result suggests that the sequential characteristics of packet traffic were better captured by the LSTM model than by the feature-based SVM classifier. Therefore, the best-performing LSTM configuration was adopted for the subsequent real-time experiments and containment evaluation.

### 3.2. Real-Time Identification of Normal and Attack Traffic

After offline training and parameter selection, the best-performing LSTM model was deployed in the edge-computing pipeline for real-time evaluation. In the deployed setting, packet records captured from the 5G communication path were continuously forwarded to the edge node, where the trained detector classified the observed traffic into one of three categories: normal traffic, TCP SYN flood traffic, and UDP flood traffic. This experiment was conducted to verify whether the selected model could maintain reliable discrimination capability after integration into the practical detection architecture rather than only under offline test conditions.

**Figure 10:**
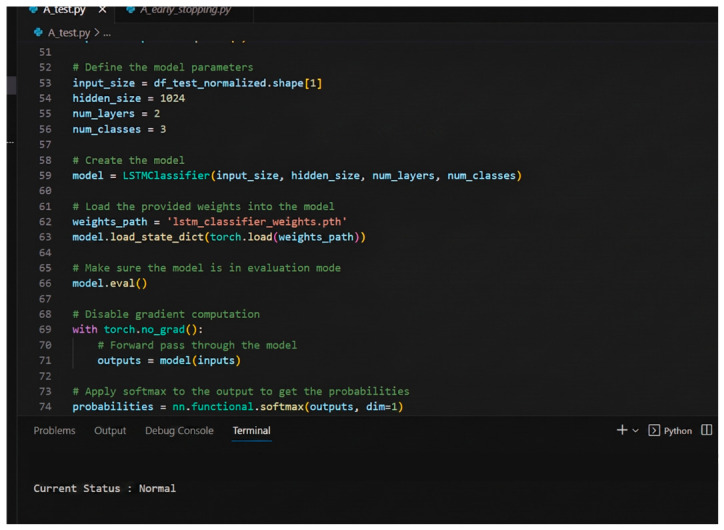
Real-time detection result for normal sensing traffic, showing that the deployed model correctly recognized benign communication during live operation.

Figure 10 presents a representative runtime result under normal operating conditions. In this case, the IoT devices transmitted regular sensing data to the web server through the 5G NSA testbed, and no attack traffic was intentionally introduced. The deployed detector identified the observed traffic as normal, indicating that legitimate sensing communication could be recognized correctly during live operation. This result supports the suitability of the deployed model for preserving normal service behavior in the experimental IoT environment.

To further simplify the presentation of the online test results, Table 7 summarizes the real-time identification outcomes under the three evaluated traffic conditions. Each traffic condition was observed for 15 min. The deployed LSTM-based detector correctly identified normal traffic, TCP SYN flood traffic, and UDP flood traffic during online operation. These results confirm that the trained model maintained its discrimination capability after being integrated into the edge-computing detection pipeline and provided a reliable basis for the subsequent authorization update process.

**Figure 11:**
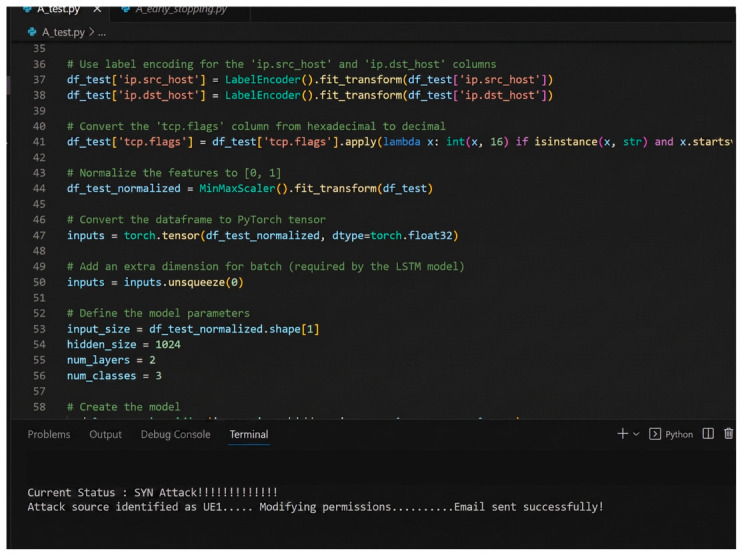
Real-time detection result for TCP SYN flood traffic, showing that the deployed model identified abnormal SYN flooding behavior during online operation.

Figure 11 presents the runtime identification result when TCP SYN flood traffic was generated by the emulated attack nodes. Under this condition, the detector processed the incoming packet stream in real time and classified the abnormal traffic as a TCP SYN flood attack. This result demonstrates that the deployed model could capture the traffic characteristics associated with SYN flooding after integration into the online pipeline and distinguish connection-oriented flooding behavior from benign sensing traffic during active testbed operation.

**Figure 12:**
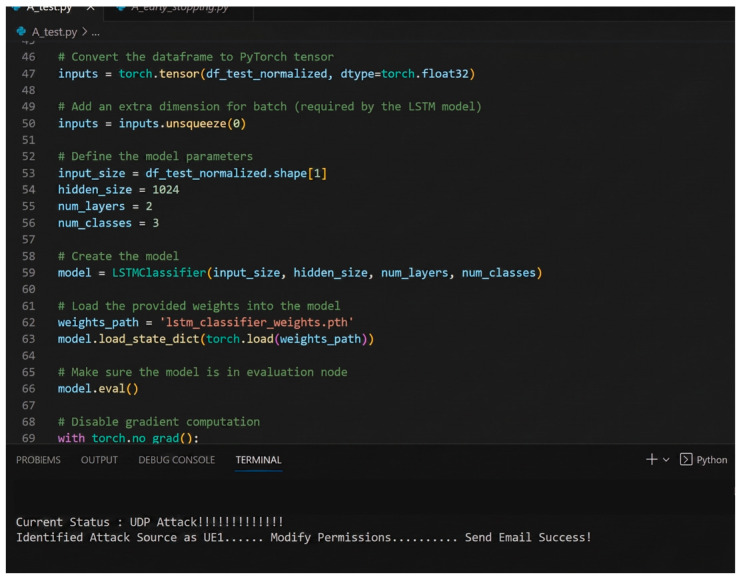
Real-time detection result for UDP flood traffic, showing that the deployed model distinguished UDP flooding traffic from normal communication during live operation.

Figure 12 presents the corresponding runtime result for the UDP flood scenario. In this experiment, malicious traffic was introduced using UDP-based flooding behavior, and the deployed detector classified the observed traffic as a UDP flood attack rather than normal communication. This result confirms that the real-time detection module was not limited to a single attack pattern and could also discriminate UDP flooding behavior during live deployment.

Taken together, Figure 10, Figure 11 and Figure 12 demonstrate that the selected LSTM detector remained effective after deployment in the edge-computing framework. The detector correctly differentiated normal sensing traffic from TCP SYN flood and UDP flood traffic during real-time operation, thereby providing the basis for the subsequent authorization update and attack-containment mechanism described in Section 3.3.

### 3.3. Attack Containment Through Zero-Trust Permission Control

Following attack identification, the abnormality report was transmitted to the authentication server, which updated the authorization table and restricted the permissions of the suspicious device. This mechanism enabled the proposed framework to integrate real-time traffic recognition with adaptive access control, thereby limiting malicious communication immediately after detection. Experimental observations showed that the framework reduced the ability of compromised devices to maintain unrestricted network access and constrained the propagation of attack traffic within the testbed. Compared with the conventional defense architecture, the zero-trust-based framework provided a more direct linkage between attack identification and containment action.

**Figure 13:**
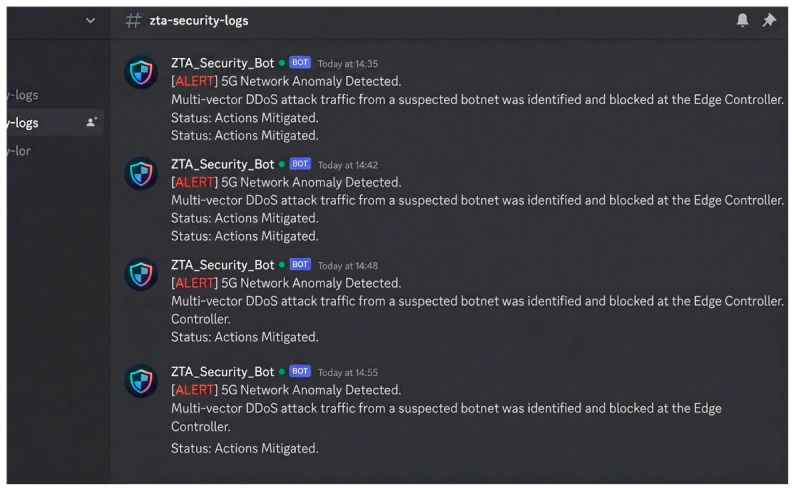
Zero-trust-based attack containment after malicious traffic detection: alert notification triggered after attack identification and permission update.

**Figure 14:**
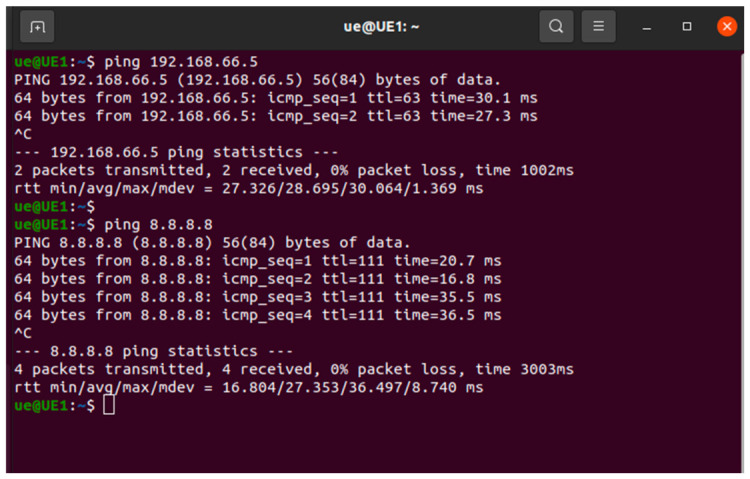
Connectivity verification result before permission restriction.

**Figure 15:**
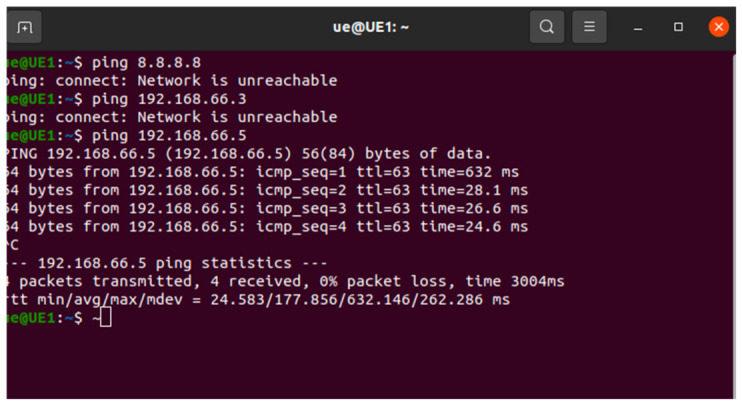
Connectivity verification result after permission restriction, showing that unauthorized external communication was blocked while controlled internal access was retained.

To clearly show how the detection results were translated into system actions, the response behavior of the proposed framework is summarized in Table 8. When normal traffic was detected, the device retained its original communication permission. In contrast, when TCP SYN flood traffic or UDP flood traffic was identified, the framework triggered an authorization update, generated an alert notification, and restricted the communication permission of the suspicious device. This result highlights that the proposed design does not stop at traffic classification, but further converts the detection outcome into an immediate containment action. The alert notification triggered after attack identification and permission update is shown in Figure 13. The connectivity verification result before permission restriction is shown in Figure 14, whereas the verification result after permission restriction is presented in Figure 15, demonstrating that unauthorized external communication was blocked while controlled internal access was preserved. 

A simplified system-level comparison between the conventional defense design and the proposed zero-trust framework is further provided in Table 9. Although both architectures support attack detection, only the proposed framework directly links the detection result to dynamic authorization update and communication restriction. In addition, the proposed design provides explicit alert notification and stronger containment of suspicious devices after attack identification. Therefore, compared with the conventional defense architecture, the proposed zero-trust-based framework provides a more direct response workflow for the evaluated 5G NSA IoT testbed.

### 3.4. Overall Findings

Overall, the experimental results show that the proposed prototype was able to perform attack detection and response under the evaluated laboratory-scale conditions. From the model comparison, the LSTM detector achieved the highest accuracy of 99.56%, which was higher than the best SVM result of 93.20%. From the real-time deployment results, the selected LSTM model correctly identified normal traffic, TCP SYN flood traffic, and UDP flood traffic under online operation. From the system response perspective, the framework further translated attack identification into authorization update, alert notification, and communication restriction for suspicious devices.

These findings indicate that the proposed design can combine intelligent traffic analysis with zero-trust-based permission control in the evaluated laboratory-scale 5G NSA IoT testbed. Rather than treating traffic detection and access control as two separate mechanisms, the proposed prototype links them within a single response workflow. As a result, the system not only detects the evaluated malicious traffic types but also supports containment action after attack identification. Therefore, the proposed framework demonstrates feasibility for improving detection-to-containment response under controlled SYN flood and UDP flood scenarios, while broader validation against more diverse DDoS variants remains necessary.

## 4. Discussion

The experimental results support the feasibility of linking packet-level DDoS detection with authorization-based response in the evaluated laboratory-scale 5G NSA IoT testbed. In the model comparison, the LSTM-based detector achieved the highest accuracy of 99.56%, whereas the best SVM configuration achieved 93.20%. This result suggests that the evaluated packet traffic contained short-term temporal patterns that were better captured by the sequence-aware LSTM model than by the feature-based SVM classifier. The real-time experiments further showed that the selected LSTM detector correctly identified normal traffic, TCP SYN flood traffic, and UDP flood traffic after being deployed in the edge-computing pipeline. Therefore, the model comparison and online test results jointly support the selection of LSTM as the detection module in this prototype.

The main contribution of this study is not limited to traffic classification accuracy. The proposed prototype further connects the detection result with a zero-trust-based permission-control mechanism. Once TCP SYN flood or UDP flood traffic was identified, the abnormality report was sent to the authentication server, which then updated the authorization table, triggered an alert notification, and restricted suspicious communication. This response workflow shows that the proposed system can translate traffic identification into a containment action within the experimental testbed. Compared with a conventional defense design that mainly detects attacks, the proposed framework provides a more direct linkage between attack identification and permission adjustment.

The current study focuses on prototype-level functional validation. Within this scope, the proposed framework implemented a detection-to-authorization workflow and demonstrated that the deployed detector could trigger permission updates and communication restriction under the evaluated traffic conditions. As summarized in Figure 1, the validation was limited to a controlled laboratory-scale 5G NSA IoT testbed and three traffic conditions: normal traffic, TCP SYN flood traffic, and UDP flood traffic. Therefore, the reported LSTM accuracy of 99.56% should be interpreted only within these evaluated conditions and should not be regarded as evidence of general robustness against all DDoS variants.

More realistic scenarios, including randomized botnet-like traffic, slow-rate attacks, adaptive packet-size variation, different inter-arrival patterns, mixed benign-malicious traffic ratios, and heterogeneous IoT workloads, were not evaluated in the current study. In addition, deployment-oriented metrics such as detection-to-containment latency, throughput, CPU/memory utilization, and scalability under large concurrent device populations were not measured. The edge-computing function was executed on a dedicated PC rather than on resource-constrained Raspberry Pi-based edge nodes. Therefore, the present results should be interpreted as controlled prototype validation rather than evidence of commercial-scale readiness.

Similarly, the implemented permission-control mechanism should be regarded as a zero-trust-based prototype rather than a complete operational implementation of Zero Trust Architecture. The current system supports registration, authentication, permission-table update, alert notification, and detection-triggered communication restriction. However, it does not yet implement a full risk-scored policy engine, continuous trust evaluation, fine-grained micro-segmentation, or component attestation. These functions should be incorporated in future work before the system can be considered a more complete zero-trust deployment.

The model and security assumptions also define the scope of the present work. SVM and LSTM were selected for comparison because SVM provides a conventional structured-feature baseline, whereas LSTM can process short packet sequences and capture temporal dependencies. However, LSTM performance depends on the quality and representativeness of the training data, and the model may not generalize well to attack types that differ substantially from the evaluated traffic. The framework also assumes that the authentication server and edge-computing node operate correctly. If these trusted components are compromised, detection outputs, authorization records, or enforcement decisions may be manipulated. These issues do not invalidate the prototype results, but they indicate that additional protection mechanisms are required before deployment in less controlled environments.

Future work should extend the current prototype in several directions. Since realistic 5G IoT deployments may involve heterogeneous devices, different traffic demands, and resource-constrained operating conditions, larger device populations, higher traffic loads, and more diverse IoT workloads should be used to evaluate scalability [27]. End-to-end detection-to-containment latency should be measured by timestamping packet capture, model inference, abnormality reporting, authorization update, and enforcement verification. More challenging attack variants, including randomized flooding, slow-rate attacks, and mixed benign-malicious traffic, should be included to evaluate model generalization. The permission-control mechanism may also be extended with risk-scored policy decisions, micro-segmentation, and component attestation. Network slicing could further support finer-grained containment by quarantining suspicious devices into dedicated slices rather than relying only on permission revocation [28]. Trust evaluation methods may also be incorporated to make permission adjustment more context-aware and adaptive in future versions of the framework [29].

## 5. Conclusions

This study presented a laboratory-scale prototype that combines real-time DDoS traffic detection with a zero-trust-based permission-control mechanism in a 5G NSA IoT testbed. Two detection models, namely SVM and LSTM, were implemented and compared for packet-level traffic classification. The experimental results showed that, under the evaluated controlled traffic conditions, the LSTM model achieved the highest accuracy of 99.56%, outperforming the best SVM result of 93.20%. These findings indicate that LSTM captured the sequential characteristics of packet-level attack traffic better than SVM under the evaluated experimental conditions.

In addition to the offline model comparison, the selected LSTM detector was deployed in the edge-computing pipeline for real-time evaluation. Under online operation, the deployed model correctly identified normal traffic, TCP SYN flood traffic, and UDP flood traffic in the 5G NSA IoT testbed. 

After abnormal traffic was detected, the detection result was transmitted to the authentication server, which dynamically updated device permissions, restricted suspicious communication, and supported administrator notification. Therefore, the proposed framework was able not only to detect malicious traffic but also to translate the detection output into an immediate containment action. Compared with the conventional defense architecture, the proposed zero-trust-based framework provided a more direct linkage between attack detection and response. This design supported the containment of suspicious devices and provided a more direct response workflow in the evaluated experimental 5G IoT environment. Overall, the results demonstrate the feasibility of combining packet-level traffic analysis with zero-trust-based permission management in the evaluated laboratory-scale 5G NSA IoT testbed. The proposed prototype successfully linked detection outputs with authorization updates and communication restriction under controlled normal, TCP SYN flood, and UDP flood traffic conditions. However, the current validation remains limited to a small-scale testbed and simple flooding scenarios. Future studies should evaluate larger device populations, more heterogeneous IoT workloads, randomized botnet-like traffic, slow-rate attacks, mixed benign-malicious traffic, and detection-to-containment latency before the framework can be generalized to broader 5G IoT deployments.

## Figures and Tables

**Table 1:** Summary of representative related studies.

Study	Environment/Dataset	Detection Method	Control/Response Mechanism	Limitation/Scope	Relation to this Study
NIST SP 800-207 [7]	General enterprise/system architecture	Not a detection model	Policy decision and policy enforcement based on zero-trust principles	Provides architectural guidance rather than DDoS detection experiments	Used as the conceptual basis for clarifying that this work implements only a limited zero-trust-based permission-control mechanism
Ramezanpour and Jagannath [10]	5G/6G networks	ML-assisted intelligent ZTA concept	Dynamic zero-trust-oriented authorization architecture	Mainly conceptual; no 5G NSA IoT DDoS testbed validation	Provides a 5G/6G-oriented ZTA reference for positioning the proposed prototype
Farzaneh et al. [18]	5G network slicing testbed	Deep transfer learning for DDoS detection	Not the main focus	Focuses on detection performance; authorization adjustment is not emphasized	Provides a stronger 5G-oriented detection reference than general IoT datasets
Bahashwan et al. [19]	SDN-related DDoS studies	Review of ML/DL/hybrid DDoS detection methods	Depends on reviewed studies	Review study; not a deployed 5G NSA IoT prototype	Shows that many DDoS studies focus on detection models and datasets
Alosaimi et al. [20]	BoT-IoT dataset	Deep learning and multi-stage IDS design	Not emphasized	Dataset-based evaluation; no 5G NSA testbed or zero-trust response	Provides comparison with IoT intrusion detection studies based on public datasets
Ali et al. [21]	SDN environment	ML/DL comparison for DDoS detection	Not emphasized	Focuses on classifier comparison and detection accuracy	Supports the use of SVM and deep learning as detection baselines
Mansoor et al. [22]	SDN controller	Deep learning-based DDoS detection	Not emphasized	Focuses on SDN controller protection; no 5G NSA IoT permission-control workflow	Provides a related example of DL-based DDoS detection in programmable networks
Banaamah and Ahmad [23]	IoT intrusion detection dataset	CNN, LSTM, and GRU	Not emphasized	Dataset-based IoT IDS evaluation; no detection-to-authorization linkage	Supports the use of sequence-aware deep learning for IoT intrusion detection
This study	Laboratory-scale 5G NSA IoT testbed	LSTM and SVM using packet-level traffic	Registration, authentication, detection-triggered permission update, alert notification, and communication restriction	Small-scale testbed; only normal, TCP SYN flood, and UDP flood traffic; no slow-rate attacks, mixed benign-malicious traffic, risk-scored policy engine, micro-segmentation, or large-scale latency/scalability evaluation	Demonstrates prototype-level feasibility of linking real-time traffic detection with authorization adjustment in a controlled 5G NSA IoT environment

**Table 2:** Experimental hardware specifications.

Device	OS	CPU	Memory	Connectivity	USB Ports
Raspberry Pi 3B (×4)	Raspberry Pi OS 64-bit	1.2 GHz ARM Cortex-A53	1 GB LPDDR2	Wi-Fi 2.4 GHz 802.11n, 10/100 RJ45, Bluetooth 4.1/BLE	4 × USB 2.0
Raspberry Pi 4B (×4)	Ubuntu Server 20.04 LTS 64-bit	1.5 GHz quad-core ARM Cortex-A72	4 GB LPDDR4	Wi-Fi 2.4/5.0 GHz 802.11b/g/n/ac, 10/100/1000 RJ45, Bluetooth 5.0/BLE	2 × USB 2.0 2 × USB 3.0
Edge PC	Windows 11	Intel i7-10700	16 GB DDR4-3200	-	-
Base-station PC	Ubuntu 20.04	Intel i7-6700	8 GB DDR3-1600	-	-
USRP B210 (×2)	-	-	-	70 MHz–6 GHz, TX×2, RX×2	USB 3.0
USRP B200 (×1)	-	-	-	70 MHz–6 GHz, TX×1, RX×1	USB 3.0

**Table 3:** Hping3 attack parameters.

Attack Type	hping3 Command Options
TCP SYN Flood	-S --flood -V -p 80
UDP Flood	--udp -c 100 --flood -V -p 53 --rand-source

**Table 4:** Accuracy of SVM under different parameter settings.

C	1	10	100
Gamma			
10	92.32%	**93.20** **%**	**93.20** **%**
100	61.16%	65.31%	65.31%
1000	45.36%	46.95%	46.95%

**Table 5:** Accuracy of LSTM under different parameter settings.

Hidden Size	1024	2048	3072
Learning Rate			
0.00001	85.70%	88.44%	83.33%
0.0001	98.15%	98.32%	98.15%
0.001	99.47%	99.56%	**99.56** **%**

**Table 6:** Summary of the best detection performance of the two models.

Model	Best Parameter Setting	Best Accuracy	Improvement Over SVM (Percentage Points)
SVM	gamma = 10, C = 10 or 100	93.20%	-
LSTM	learning rate = 0.001, hidden size = 2048 or 3072	99.56%	6.36

**Table 7:** Summary of real-time identification results under different traffic conditions.

Traffic Condition	Observation Duration	Detection Output	Identification Result
Normal traffic	15 min	Normal	Correct
TCP SYN flood traffic	15 min	TCP SYN flood	Correct
UDP flood traffic	15 min	UDP flood	Correct

**Table 8:** Response actions of the proposed framework after traffic identification.

Detected Traffic Type	Authorization Update	Alert Notification	Communication Restriction
Normal traffic	No	No	No
TCP SYN flood traffic	Yes	Yes	Yes
UDP flood traffic	Yes	Yes	Yes

**Table 9:** System-level comparison between the conventional defense design and the proposed zero-trust framework.

Item	Conventional Defense Architecture	Proposed Zero-Trust Framework
Real-time attack detection	Yes	Yes
Dynamic authorization update	No	Yes
Alert notification after attack identification	Limited	Yes
Restriction of suspicious device communication	Limited	Yes
Attack propagation containment	Weaker	Stronger

## Data Availability

The data underlying this article cannot be shared publicly in order to maintain confidentiality and protect ongoing research. Researchers interested in accessing the dataset must submit a request to the corresponding author.
